# A Paradoxical Case of Bleeding and Clots: The Hidden Thrombotic Threat in Immune Thrombocytopenic Purpura

**DOI:** 10.1002/ccr3.73564

**Published:** 2026-09-21

**Authors:** Nayon Kumar Sarker, Sudipta Das, Md. Kamrul Alam, Sreyoshi Dutta Sarker, Md. Habibur Rahman

**Affiliations:** ^1^ Chittagong Medical College Hospital Chattogram Bangladesh; ^2^ Bangabandhu Sheikh Mujib Medical University Dhaka Bangladesh

**Keywords:** case report, deep vein thrombosis (DVT), eltrombopag, immune thrombocytopenic purpura (ITP), norethisterone, pulmonary embolism

## Abstract

Immune thrombocytopenic purpura (ITP) is typically characterized by mucocutaneous bleeding, yet paradoxical thrombotic events are increasingly recognized, particularly in patients treated with thrombopoietin‐receptor agonists (TPO‐RAs) such as eltrombopag. We describe a young woman with established ITP who developed deep vein thrombosis and pulmonary embolism shortly after commencing norethisterone for menstrual regulation while also using eltrombopag, creating a combination of therapeutic and physiological prothrombotic risks. Despite persistently low platelet counts, she presented with acute dyspnea, pleuritic chest pain, and unilateral lower limb swelling. CT pulmonary angiography demonstrated segmental pulmonary emboli, and Doppler ultrasonography confirmed lower limb thrombosis. Both eltrombopag and norethisterone were discontinued, and anticoagulation was initiated under close hematological supervision, resulting in clinical and radiological improvement with stabilization of platelet counts on follow‐up. This case underscores the complex interplay between ITP, platelet‐stimulating agents, and hormonal therapy, illustrating how thrombosis may occur even in the context of thrombocytopenia. It highlights the importance of maintaining a high index of suspicion for thromboembolic complications in ITP patients receiving TPO‐RA therapy, particularly when additional risk factors coexist, and reinforces the need for careful risk–benefit assessment when initiating or continuing agents that may contribute to thrombogenesis.

**Key Clinical Message**

Eltrombopag, a thrombopoietin‐receptor agonist (TPO‐RA), may precipitate thrombotic events in patients with immune thrombocytopenic purpura (ITP) despite thrombocytopenia, and the concomitant use of hormonal agents like norethisterone may further amplify the risk. Therefore, co‐prescribing these agents should be approached with careful clinical consideration.

## 1 Introduction

Immune thrombocytopenic purpura (ITP) is an autoimmune disorder characterized by isolated thrombocytopenia resulting from diminished platelet production and increased platelet destruction [1, 2]. Although the condition is classically associated with bleeding, emerging evidence suggests an increased risk of thrombosis, particularly following the use of thrombopoietin receptor agonists (TPO‐RAs) such as eltrombopag [2, 3, 4, 5]. Simultaneous administration of hormonal agents, such as norethisterone, may further increase this risk [6]. Here, we describe a rare but clinically significant case of deep vein thrombosis (DVT) and pulmonary embolism (PE) in an ITP patient on eltrombopag and norethisterone therapy.

## 2 Case History/Examination

A 26‐year‐old woman with a 6‐year history of ITP presented with progressive swelling of the right lower limb for 10 days and shortness of breath for 7 days. She also complained of sudden, sharp, and non‐radiating chest pain for the same duration with occasional hemoptysis. Her initial diagnosis of ITP was based on clinical findings (e.g., gum bleeding, purpura, and menorrhagia) and bone marrow examination in 2019. She had been under hematology follow‐up and was started on eltrombopag 3 months before presentation. She was prescribed norethisterone (5 mg thrice daily) for menstrual irregularity while already taking eltrombopag. Notably, about 2 weeks after starting norethisterone, she developed swelling of the right leg accompanied by pleuritic chest pain, dyspnea, and hemoptysis. She was subsequently referred to our tertiary center for evaluation. There was no prior history of thrombotic events or hereditary thrombophilia.

**FIGURE 1:**
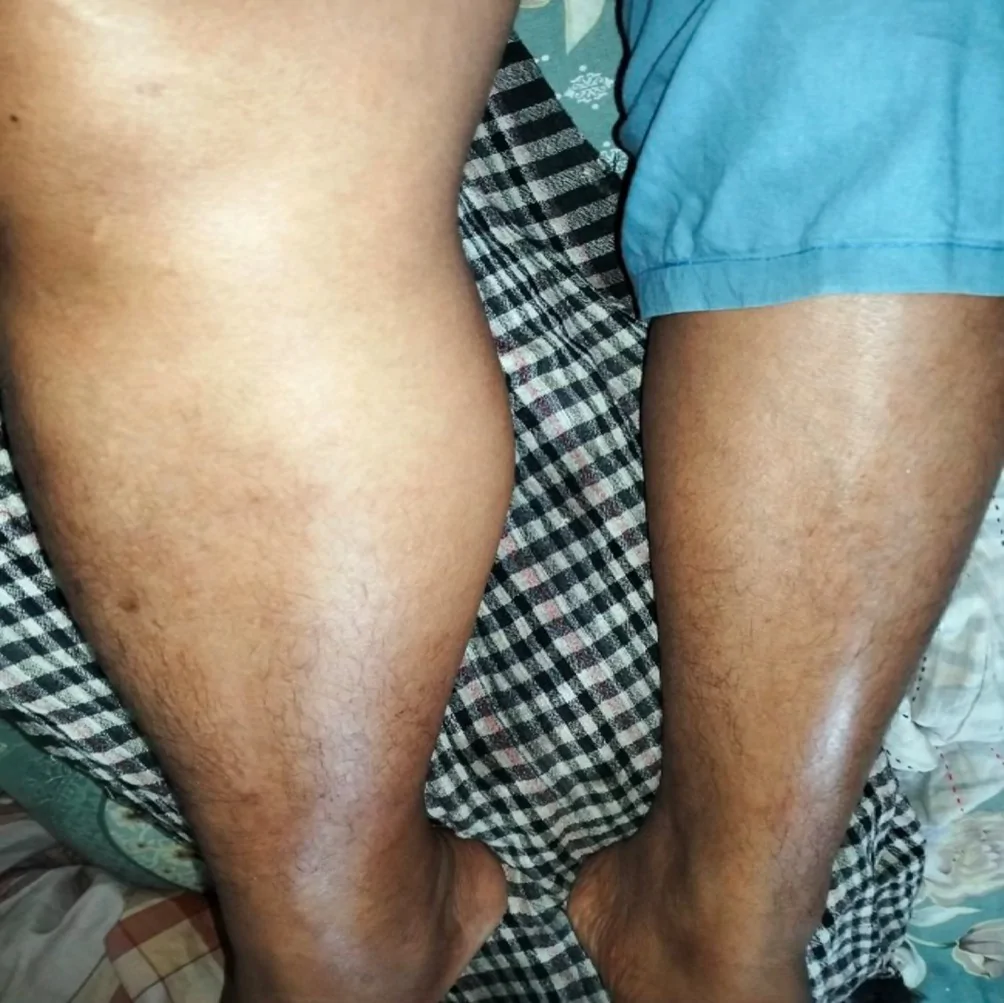
Clinical image showing right lower limb swelling consistent with deep vein thrombosis (DVT).

On examination, the patient was mildly hypoxic (SpO_2_ 93% on face mask), tachypneic (RR 24/min), and tachycardic (HR 110 bpm). Her blood pressure was 100/60 mmHg, and her temperature was 98°F. There was pitting edema, redness, and warmth in the right lower limb, with a calf circumference difference of > 3 cm. There was tenderness along the deep veins (Figure 1).

## 3 Differential Diagnosis, Investigations, and Treatment

The following differential diagnoses were considered:

1. Immune thrombocytopenic purpura with drug‐induced thrombosis.
2. Primary antiphospholipid syndrome.
3. Inherited thrombophilia.

Given the patient's young age and venous thromboembolism, primary antiphospholipid syndrome was evaluated. An antiphospholipid antibody panel, including lupus anticoagulant, anticardiolipin antibodies (IgG and IgM), and anti‐β2 glycoprotein I antibodies (IgG and IgM), was negative, thereby excluding antiphospholipid syndrome.

Inherited thrombophilia was considered; however, in the presence of a clear provoking factor related to recent exposure to eltrombopag and norethisterone, and in the absence of any personal or family history of venous thromboembolism, inherited thrombophilia was considered less likely, and further thrombophilia testing was not pursued.

**FIGURE 2:**
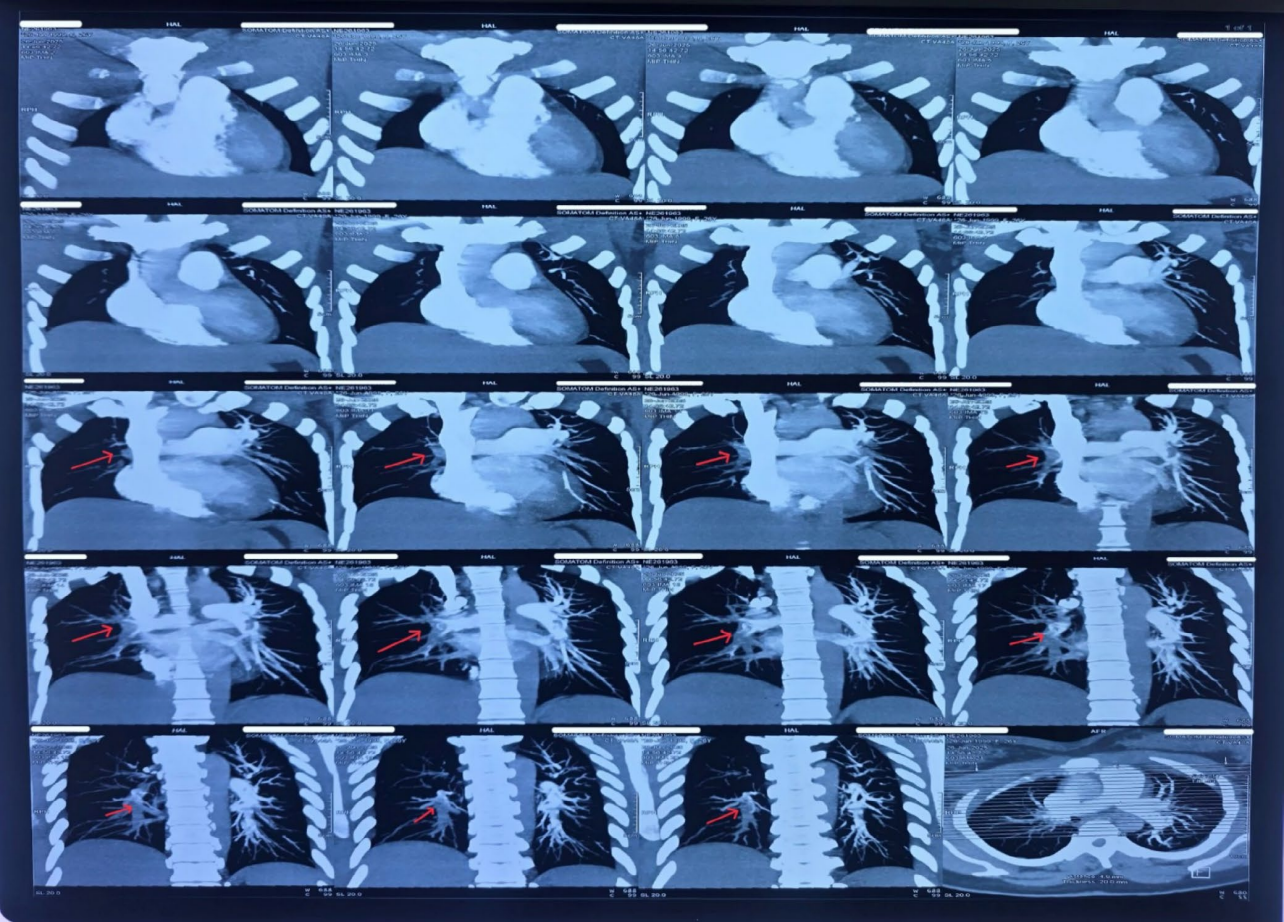
The CT pulmonary angiogram shows a distal filling defect in the right pulmonary artery, consistent with pulmonary embolism.

Laboratory findings revealed a platelet count of 109 × 10^9^/L and elevated D‐dimer levels. CT pulmonary angiogram showed a distal filling defect in the right pulmonary artery (Figure 2).

**FIGURE 3:**
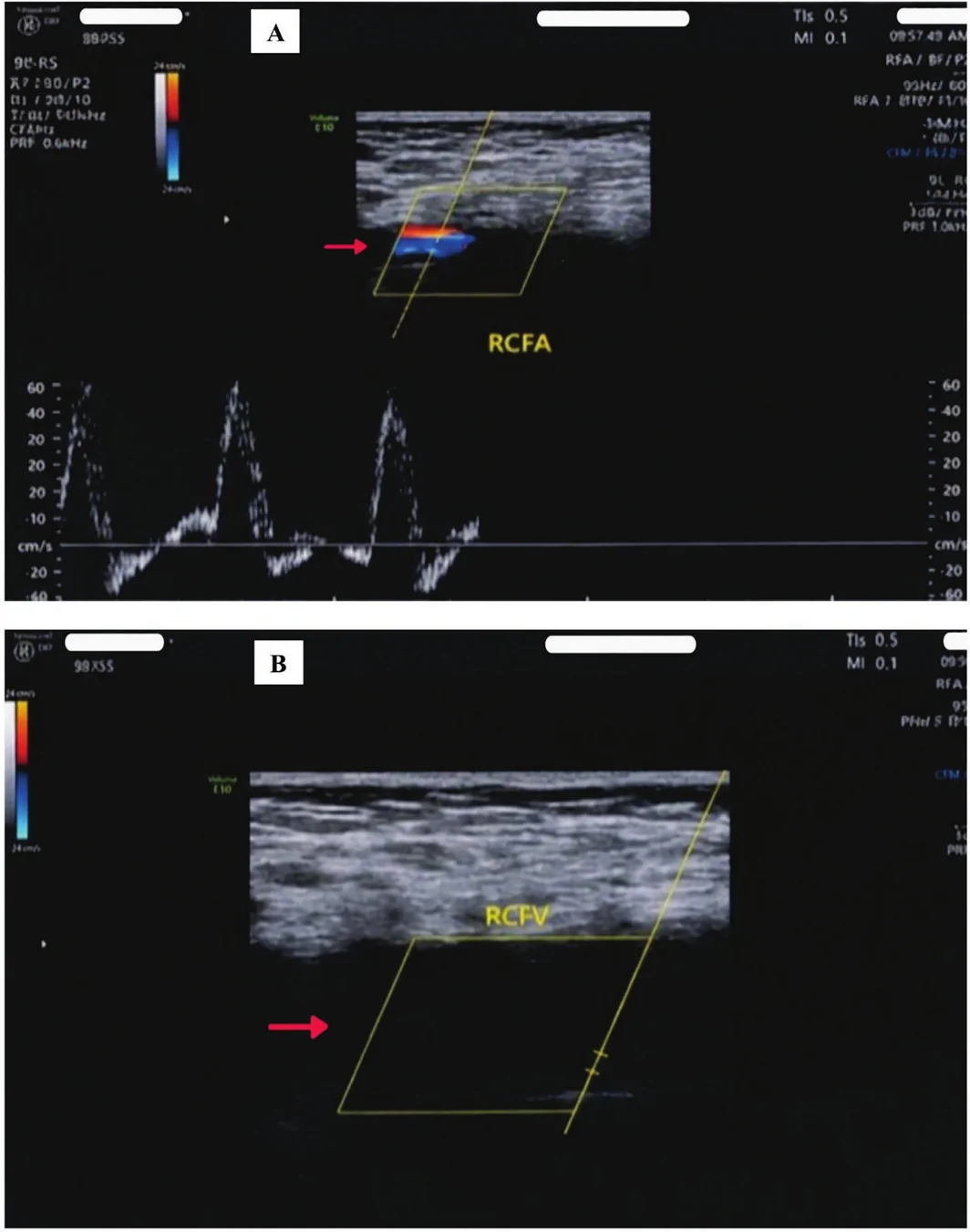
Color Doppler ultrasound of right lower limb vessels. (A) The color Doppler ultrasound of the right common femoral artery shows a triphasic waveform consistent with normal flow. (B) The color Doppler ultrasound of the right common femoral vein showing dilated, non‐compressible, and no color flow, consistent with thrombosis.

Color Doppler ultrasound of the right common femoral vein (RCFV) found it noncompressible with reduced flow (Figure 3).

With the above findings, a diagnosis of drug‐induced DVT and PE in this patient with chronic ITP was made. Eltrombopag and norethisterone were immediately discontinued. The patient was started on therapeutic low molecular weight heparin (enoxaparin) and later transitioned to apixaban.

## 4 Conclusion and Results (Outcome and Follow‐Up)

Over 1 week, she showed significant clinical improvement and was discharged in stable condition. A follow‐up imaging showed resolution of the thrombus. Platelet counts remained stable off eltrombopag.

## 5 Discussion

This case report presents the history of a young female in her mid‐twenties suffering from chronic, primary immune thrombocytopenic purpura (ITP) who developed thrombosis following eltrombopag and norethisterone therapy.

Thrombosis in ITP is paradoxical but increasingly recognized [2, 5, 7]. Several risk factors have been identified, including increasing age, male gender, secondary ITP, a history of thrombosis, and the line of therapy, particularly after thrombopoietin receptor agonist therapy (TPO‐RA) [2, 7]. Although clinical trials have not identified which thrombopoietin receptor agonist therapy poses a substantial risk of thrombosis, several cases of eltrombopag‐induced thrombosis have been reported worldwide [8, 9, 10, 11, 12]. Eltrombopag stimulates megakaryocyte proliferation and platelet production, which may result in increased platelet activation and procoagulant activity [11]. In our patient, the use of eltrombopag was considered an important potential risk factor for developing thrombosis, which was further compounded by exposure to another potential prothrombotic agent, norethisterone, a synthetic progestin commonly used for menstrual irregularities. Some previous evidence has found norethisterone to be a potential gold standard contraceptive; considering the risk of thrombosis, reports show that hormonal therapy, specifically progestins like norethisterone, independently contributes to hypercoagulability by increasing clotting factor synthesis, reducing fibrinolysis, and increasing platelet activation [6, 13, 14].

In this case, the thrombotic episode occurred approximately 2 weeks after starting norethisterone while already on eltrombopag. This temporal association raises the possibility that both medications had an additive prothrombotic effect in the development of thromboembolism. The patient lacked other known risk factors such as recent immobilization, surgery, malignancy, or positive family history of thrombotic events. The antiphospholipid antibody panel was negative, and comprehensive inherited thrombophilia testing was not performed during this clinical evaluation. Considering the acute episode of thrombosis, testing for natural anticoagulant deficiencies, such as protein C, protein S, and antithrombin, may be unreliable during the acute phase. Current guidance recommends that, when clinically indicated, testing for these deficiencies be performed after the acute phase of thrombosis and after completing an appropriate period of anticoagulation because early results may be unreliable [15]. Given the temporal relationship between potential provoking agents (eltrombopag and norethisterone) and the subsequent thrombotic event, additional inherited thrombophilia testing was not performed during this clinical evaluation.

Several worldwide studies have described thrombotic complications associated with TPO‐RAs in patients with ITP [8, 9, 10, 11, 12]. Ghumman et al. [10] reported a case of deep vein thrombosis with bilateral pulmonary embolism following eltrombopag therapy, highlighting the potential thrombotic risk associated with TPO‐RA use. Similarly, eltrombopag‐associated cerebral venous thrombosis has been reported in patients with ITP, emphasizing that thrombotic events may occur at unusual vascular sites despite thrombocytopenia [12]. Oo et al. [9] further described eltrombopag‐associated thrombosis in patients with ITP and antiphospholipid syndrome, suggesting that additional prothrombotic factors may further increase thrombotic risk during TPO‐RA therapy. Compared with these reports, our patient developed both DVT and pulmonary embolism shortly after the initiation of norethisterone while receiving eltrombopag, with a negative antiphospholipid antibody panel and no other documented thrombotic risk factors. Although causality cannot be established from this single case, this temporal association suggests a possible additive prothrombotic effect of concurrent TPO‐RA (e.g., eltrombopag) and hormonal therapy (e.g., norethisterone) and highlights the importance of individualized thrombotic risk assessment.

Our case underscores the need for individualized therapy balancing bleeding versus clotting risk. In patients achieving safe platelet counts, dose adjustment or temporary cessation of TPO‐RA therapy may be considered when concurrent use of another potential prothrombotic agent is required to reduce potential thrombotic complications.

## Author Contributions

**Nayon Kumar Sarker:** conceptualization, data curation, formal analysis, investigation, methodology, visualization, writing – original draft, writing – review and editing. **Sudipta Das:** conceptualization, methodology, visualization, writing – original draft, writing – review and editing. **Md. Kamrul Alam:** conceptualization, methodology, visualization, writing – original draft, writing – review and editing. **Sreyoshi Dutta Sarker:** conceptualization, methodology, visualization, writing – original draft, writing – review and editing. **Md. Habibur Rahman:** conceptualization, methodology, supervision, validation, writing – review and editing.

## Funding

The authors have nothing to report.

## Ethics Statement

The authors have nothing to report.

## Consent

Written informed consent was obtained from the patient to publish the case report and any accompanying images.

## Conflicts of Interest

The authors declare no conflicts of interest.

## Data Availability

This submitted manuscript includes all the data generated or analyzed during this study relevant to this case.
